# Unexpected Primary Spontaneous Pneumothorax in a Non-smoking Patient After Blowing Out Birthday Candles

**DOI:** 10.7759/cureus.92755

**Published:** 2025-09-19

**Authors:** Leila Laouar, Nadia Dammene Debbih

**Affiliations:** 1 Faculty of Medicine, Youcef El Khatib University of Health Sciences, Algiers, DZA; 2 Department of Pulmonology, Mustapha University Hospital Center, Algiers, DZA; 3 Department of Cardiology, Mustapha University Hospital Center, Algiers, DZA

**Keywords:** chest tube drainage, long-term outcome, primary spontaneous pneumothorax, tall and slim morphology, trivial respiratory effort

## Abstract

Primary spontaneous pneumothorax (PSP) is defined as the presence of air in the pleural cavity in the absence of underlying pulmonary disease. It predominantly affects young, tall, slim males, who are often smokers. However, it can also occur in healthy non-smokers without comorbidities. Sudden respiratory efforts, although rarely reported, may act as triggering factors. We report the case of a 20-year-old non-smoking man with a slender build and no medical history, who experienced acute left-sided chest pain while blowing out birthday candles. Clinical examination revealed subtle signs of pleural air. A chest X-ray confirmed a left-sided PSP without mediastinal shift. Initial conservative management was started, but due to clinical worsening, chest tube drainage was performed. The outcome was favorable, with no recurrence observed after five years of follow-up. This case illustrates an atypical form of PSP occurring in a young, non-smoking, slim individual following a trivial respiratory effort. It highlights the importance of recognizing everyday actions as potential triggers and ensuring appropriate management and long-term follow-up to prevent recurrence.

## Introduction

Primary spontaneous pneumothorax (PSP) is defined as the accumulation of air in the pleural space without any apparent cause or underlying pulmonary pathology. It is distinguished from secondary spontaneous pneumothorax (SSP), which occurs in patients with preexisting lung diseases such as chronic obstructive pulmonary disease (COPD), diffuse cystic lung diseases, silicosis, or interstitial lung disorders. [1]

PSP is a relatively common condition, predominantly affecting young, tall, thin males [2]. Smoking is a major risk factor for PSP [3]. Chest imaging frequently reveals subpleural blebs or bullae, which are implicated in the pathophysiology [4]. However, the role of blebs as the sole cause of PSP is debatable. Peripheral airway obstruction with air trapping is also thought to contribute to its pathogenesis. Although it usually occurs at rest, spontaneous pneumothorax can be triggered by sudden increases in intrathoracic pressure, such as during laughter, coughing, singing, or other abrupt respiratory efforts [5]. These mechanisms may facilitate the rupture of subpleural bullae.

The management of PSP has evolved toward more conservative and outpatient approaches, including simple aspiration or clinical observation, depending on the pneumothorax size and the patient's tolerance [6]. Recurrence is common in PSP, which may justify definitive surgical intervention, including pleurodesis or bullectomy, in selected cases.

We report here an unusual case of PSP in a tall, slender young man, occurring after a sudden respiratory effort while blowing out birthday candles. This case highlights the importance of recognizing seemingly trivial daily actions as potential triggers for PSP.

## Case presentation

Clinical presentation

A 20-year-old male university student, a non-smoker with no significant past medical history, presented to the emergency department with acute left-sided chest pain that occurred abruptly while blowing out birthday candles. The pain was rated 5 out of 10 on a visual analogue scale (VAS), pleuritic in nature, described as sharp and stabbing, and worsened by deep inspiration. It was associated with moderate dyspnea and a dry cough, painful upon expectoration.

Physical examination

Upon arrival, the patient was alert, anxious, and eupneic (respiratory rate: 20 breaths per minute) with an oxygen saturation of 95% on room air, a heart rate of 110 beats per minute, and a temperature of 37°C. His weight was 71 kg, height 1.87 m, corresponding to a body mass index (BMI) of 20.3 kg/m², indicating a slender morphotype. Pulmonary auscultation revealed a slight decrease in breath sounds and vocal fremitus on the left hemithorax, with no signs of respiratory distress or cyanosis.

Investigations

**Figure 1:**
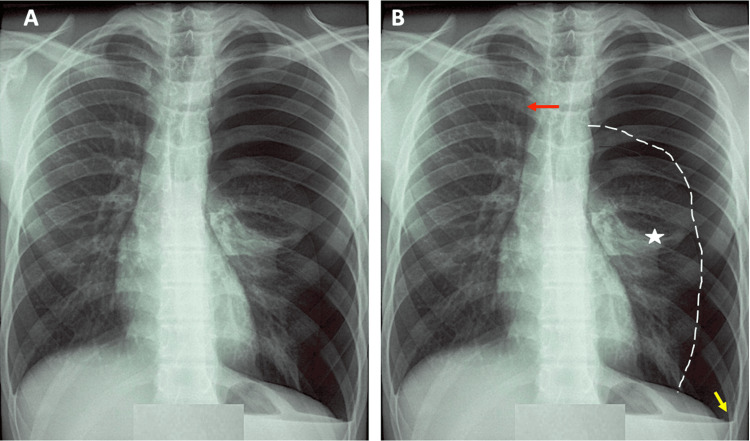
Frontal chest radiograph (A) A large avascular hyperlucent area occupies nearly the entire left hemithorax, consistent with a complete left-sided pneumothorax. (B) The left lung is completely collapsed and retracted toward the mediastinum (white star), delineated by a fine white pleural demarcation line. Blunting of the left costophrenic angle is observed (yellow arrow), suggesting associated pleural thickening or effusion. The trachea is deviated to the right (red arrow), indicating a mass effect consistent with tension physiology. The right lung appears radiographically normal. These findings are diagnostic of a compressive total left pneumothorax.

Electrocardiogram (ECG), routinely performed in the context of left-sided chest pain, demonstrated a regular sinus rhythm with no repolarization abnormalities. A posteroanterior chest X-ray taken during full inspiration revealed increased radiolucency of the left hemithorax, consistent with a pleural air collection, without fluid effusion, and retraction of the visceral pleura. The pulmonary parenchyma appeared normal on both sides, and no mediastinal shift was observed. The diagnosis of a left-sided PSP was established (Figure 1).

Therapeutic management

The patient received low-flow oxygen therapy (0.5-1 L/min) and was closely monitored clinically. Due to worsening pain (VAS: 7/10) and increased pleural detachment on follow-up imaging, a chest tube was inserted. The procedure was successful, leading to rapid clinical improvement, pain resolution, increased oxygen saturation, and complete pulmonary re-expansion confirmed on repeat chest X-ray.

Outcome and follow-up

The patient was hospitalized for three days. No complications were observed. He remained asymptomatic after chest tube removal. He was educated about preventive measures, signs of recurrence, and activity restrictions (physical exertion, air travel, etc.). No recurrence was reported after five years of follow-up.

## Discussion

PSP typically occurs in young, male individuals [7], often exhibiting a slender or elongated body habitus [8], and in the absence of any apparent underlying lung disease. The tall and thin morphotype of our patient further supports the physiopathological plausibility of this presentation. PSP is more common in individuals who are tall, slim, and have a low BMI. Tan et al. identified a correlation between low BMI and increased risk of PSP recurrence [9]. Nakamura et al. demonstrated that, in men, the weight-to-height ratio, rapid linear growth, and insufficient weight gain during adolescence are key risk features [10].

Nonomura et al. reported age-related morphological differences among PSP patients; adolescents tend to have lower BMI and a slimmer build compared to young adults [8]. While thoracic dimensions in patients in their 20s correlate proportionally with height, this correlation is less evident in adolescents. These findings suggest that anatomical variations related to age may influence PSP onset, underscoring the need for age-adapted clinical assessment. A long and narrow thorax, characterized by elongated limbs and reduced chest width, may lead to higher negative pleural pressures in the apices, thus predisposing to the formation of subpleural blebs or emphysematous bullae.  Moreover, the absence of overt lung disease does not imply structurally normal lungs. Multiple histological studies in surgically treated PSP patients have revealed subclinical abnormalities, such as distal airway inflammation, respiratory bronchiolitis, pleural thickening, or increased porosity of the visceral pleura. In our case, although the patient was a non-smoker and had a BMI within normal range (20.3 kg/m² for 1.87 m and 71 kg), his morphotype was clearly slender, consistent with the phenotypic risk factors associated with PSP.

The assumption that the underlying lung in PSP is structurally normal remains controversial. Indeed, PSP occurs predominantly (~90%) in tobacco smokers. Both cigarette smoking and cannabis use contribute to airway inflammation and the development of subclinical lung disease, notably apical bullous emphysema [11]. The critical role of smoking in the pathogenesis of PSP remains uncertain, as PSP can also develop in non-smokers. Chen et al. observed no significant differences between smokers and non-smokers in terms of clinical characteristics or radiological presence of bullae; however, smokers exhibited more extensive respiratory bronchiolitis, greater pigmentation from tobacco exposure, and significantly higher pneumothorax recurrence rates [12].

The pathophysiological mechanism of PSP is still poorly understood. Some authors suggest that a sudden increase in intra-alveolar pressure may induce the rupture of subpleural blebs, leading to air leakage into the pleural space [13-15]. Blebs are typically defined as small subpleural air-filled protrusions of the visceral pleura caused by interstitial air dissection. These lesions, formed between the internal and external elastic laminae of the pulmonary pleura, are not in direct communication with the respiratory tree (including alveoli). According to most authors, their diameter is <10 mm, though some define them as <20 mm [16]. Distal airway inflammation appears to be the initiating event in this pathogenesis. Microscopic abnormalities have been identified in up to 88.6% of lung specimens from patients undergoing surgery for PSP, typically showing chronic distal airway inflammation with lymphocyte and macrophage infiltration and fibrotic changes [17]. Although our patient was a non-smoker, a subclinical alteration of the pleural architecture cannot be excluded, which may have predisposed him to spontaneous rupture under minimal mechanical stress. This discrepancy between apparent clinical normality and microstructural abnormalities underscores the need for a deeper understanding of so-called "idiopathic" forms of PSP.

PSP often appears in clusters, possibly linked to variations in local meteorological conditions, particularly atmospheric pressure fluctuations. Several studies have confirmed this association, including a systematic review by Marx et al. [18] examining the impact of air pollution and meteorological variables on PSP incidence, and a study by Park et al. [19] demonstrating an increased risk of PSP-related emergency department visits in the presence of air pollutants and low atmospheric pressure. 

In addition to environmental factors, sudden respiratory efforts, sometimes seemingly trivial, should also be considered in understanding PSP onset. Although these efforts are often minor, they may generate significant mechanical stress on structurally weakened pulmonary areas. PSP occurs most frequently at rest or during low-intensity activities (70%), during sleep (16%), and less commonly in relation to physical exertion (3%) [20]. This distribution highlights the frequent occurrence of PSP in the absence of physical effort, which can complicate the identification of immediate causes. According to Weissberg and Refaely, only 10% of PSP episodes occur following physical exertion [21]. While physical effort has long been suspected as a contributing factor, this association has not been definitively proven.

Recent reports have described numerous cases of pneumothorax associated with seemingly innocuous daily life activities. Several cases have been described following unusual respiratory maneuvers: intense coughing [22], prolonged singing or vomiting [23], and playing wind instruments such as the trumpet, clarinet, or saxophone [24]. Playing high notes has been shown to simulate a Valsalva maneuver, as demonstrated in tuba players, potentially resulting in elevated intra-alveolar pressures and pneumothorax via the Macklin effect. Macklin et al. first described the mechanism of pneumomediastinum and subcutaneous emphysema due to alveolar rupture caused by a large pressure gradient against a closed glottis [25]. Professional musicians may be susceptible to PSP due to bleb rupture associated with repeated alveolar overdistension and subsequent air leakage into the pleural cavity once a critical pressure is reached [26].

Although rarely reported, the association between PSP and exposure to high sound levels has been suggested in the literature. Noppen et al. reported five PSP episodes in four patients exposed to strong acoustic vibrations at concerts or nightclubs, without active instrumental involvement at the time of symptom onset [27]. In the cases reported by them, the first patient experienced acute right-sided chest pain during a pop music concert; the second developed left-sided chest pain in a nightclub while standing next to a powerful speaker. The third patient experienced two separate PSP episodes, both during heavy metal concerts, while the fourth suffered a PSP recurrence while listening to extremely loud music inside his car. These clinical observations suggest that intense acoustic vibrations, particularly low-frequency pressure waves transmitted through the chest wall, may transiently increase intrathoracic pressure. In predisposed individuals, especially those with subpleural blebs or bullae, this increase may suffice to induce rupture and lead to PSP.

A similar mechanism may occur in situations involving extremely high oral pressures, such as inflating multiple balloons in a short period [24] or during mild physical efforts like lifting light weights, performing certain yoga breathing exercises [28], or simply blowing out birthday candles. In our reported case, deep inspiration alone was sufficient to trigger PSP, particularly in an individual with underlying pulmonary architectural fragility. Early identification of such often-overlooked and seemingly trivial triggers is essential for effective patient education, recurrence prevention, and therapeutic decision-making. A meticulous clinical history, particularly regarding the context of symptom onset, even in the absence of overt effort, should be emphasized in clinical practice.

The diagnosis of PSP is based on clinical assessment and imaging studies. Patients typically present with acute, pleuritic chest pain on the affected side, acute dyspnea with increased respiratory effort, and sometimes a dry, painful cough. Ghisalberti et al. reported chest pain in 69.25% of patients (range 9% to 100%) and dyspnea in 54.55% (range 27% to 77.1%) [29].

In some cases, PSP can be completely asymptomatic. The risk was higher in males, particularly those who were taller, had a lower BMI, were younger, and exhibited rapid linear growth. Among patients with mild lung collapse (<10%), a notable proportion may ultimately require invasive treatment [30]. These findings highlight the importance of early detection, close follow-up, and consideration of rapid somatic growth as a risk factor for PSP, even in the absence of symptoms. Tension pneumothorax is a rare but potentially life-threatening complication of spontaneous pneumothorax. It typically presents with delayed signs such as hypoxemia, jugular venous distension, paradoxical pulse, hypotension, and tracheal deviation.

Chest radiography remains the initial diagnostic tool of choice. It typically shows displacement of the visceral pleural line with the absence of lung markings between the pleural line and the chest wall. Upright posteroanterior (PA) radiographs are preferred, although expiratory or lateral decubitus views do not significantly enhance diagnostic sensitivity. Thoracic ultrasound is increasingly recognized as a valuable diagnostic tool. It allows direct visualization of the pleural layers as a thin hyperechoic line (<2 mm) [31]. Ultrasound is also helpful for monitoring patients with chest drains and distinguishing between subpleural blebs and pneumothorax.  Chest computed tomography (CT) is indicated when the diagnosis remains uncertain or if initial imaging is equivocal. It offers excellent sensitivity [32] and allows for accurate estimation of pneumothorax volume [33]. High-resolution CT (HRCT) can detect subpleural blebs or bullae associated with PSP and can reveal rare cystic lung diseases [32]. 

The management of PSP relies on a stepwise strategy based on the patient's clinical, radiological, and contextual characteristics. Therapeutic options include simple observation, oxygen therapy, needle aspiration, chest tube drainage, and secondary prevention measures such as surgery or pleurodesis in complex or recurrent cases [34].

## Conclusions

This case highlights the importance of jointly considering two key elements in the occurrence of PSP. Morphological predisposition, particularly a tall and slender body habitus, is recognized as a vulnerability factor even in the absence of smoking, the main risk factor reported. Besides these, there may be atypical triggering factors, often underestimated, but capable of inducing pleural rupture in predisposed individuals. These observations underscore the need to educate patients with such a morphotype about the clinical warning signs of PSP, such as sudden chest pain or acute dyspnea, in order to ensure early and appropriate management. Moreover, this case reinforces the importance of a thorough medical history, including environmental or mechanical triggers, in the diagnostic assessment of PSP occurring in young individuals with no apparent underlying pulmonary disease.

## References

[1] Huan NC, Sidhu C, Thomas R (2021). Pneumothorax: classification and etiology. Clin Chest Med.

[2] Mummadi SR, Stoller JK, Lopez R (2021). Epidemiology of adult pleural disease in the United States. Chest.

[3] Kim D, Eom SY, Shin CS, Kim YD, Kim SW, Hong JM (2020). The clinical effect of smoking and environmental factors in spontaneous pneumothorax: a case-crossover study in an Inland province. Ther Adv Respir Dis.

[4] Martínez-Ramos D, Angel-Yepes V, Escrig-Sos J (2007). Usefulness of computed tomography in determining risk of recurrence after a first episode of primary spontaneous pneumothorax: therapeutic implications. Arch Bronconeumol.

[5] Sahn SA, Heffner JE (2000). Spontaneous pneumothorax. N Engl J Med.

[6] Plojoux J, Froudarakis M, Janssens JP, Soccal PM, Tschopp JM (2019). New insights and improved strategies for the management of primary spontaneous pneumothorax. Clin Respir J.

[7] Bobbio A, Dechartres A, Bouam S (2015). Epidemiology of spontaneous pneumothorax: gender-related differences. Thorax.

[8] Nonomura R, Sugawara T, Yabe R, Oshima Y, Sasaki T, Ishibashi N (2024). Age-specific body shape characteristics in the onset of spontaneous pneumothorax: a comparison between teens and 20s. Cureus.

[9] Tan J, Yang Y, Zhong J (2017). Association between BMI and recurrence of primary spontaneous pneumothorax. World J Surg.

[10] Nakamura H, Izuchi R, Hagiwara T (1983). Physical constitution and smoking habits of patients with idiopathic spontaneous pneumothorax. Jpn J Med.

[11] Ruppert AM, Perrin J, Khalil A (2018). Effect of cannabis and tobacco on emphysema in patients with spontaneous pneumothorax. Diagn Interv Imaging.

[12] Cheng YL, Huang TW, Lin CK, Lee SC, Tzao C, Chen JC, Chang H (2009). The impact of smoking in primary spontaneous pneumothorax. J Thorac Cardiovasc Surg.

[13] Walker SE (1965). Spontaneous pneumothorax. Br Med J.

[14] Hallifax R (2022). Aetiology of primary spontaneous pneumothorax. J Clin Med.

[15] Yang HC, Jung S (2020). Bullae formation hypothesis in primary spontaneous pneumothorax. J Thorac Dis.

[16] (2025). National Center for Biotechnology Information: Pulmonary bleb. https://www.ncbi.nlm.nih.gov/medgen/341774.

[17] Cottin V, Streichenberger N, Gamondès JP, Thévenet F, Loire R, Cordier JF (1998). Respiratory bronchiolitis in smokers with spontaneous pneumothorax. Eur Respir J.

[18] Marx T, Bernard N, Kepka S, Gérazime A, Mauny F, Desmettre T (2021). Pneumothorax and the environment: a systematic review of the impact of air pollution and meteorology, and a meta-analysis on meteorology factors. Environ Pollut.

[19] Park JH, Lee SH, Yun SJ (2018). Air pollutants and atmospheric pressure increased risk of ED visit for spontaneous pneumothorax. Am J Emerg Med.

[20] Olesen WH, Titlestad IL, Andersen PE, Lindahl-Jacobsen R, Licht PB (2019). Incidence of primary spontaneous pneumothorax: a validated, register-based nationwide study. ERJ Open Res.

[21] Weissberg D, Refaely Y (2000). Pneumothorax: experience with 1,199 patients. Chest.

[22] Zatovkaňuková P, Slíva J (2024). The potential dangers of whooping cough: a case of rib fracture and pneumothorax. BMC Infect Dis.

[23] Forshaw MJ, Khan AZ, Strauss DC, Botha AJ, Mason RC (2007). Vomiting-induced pneumomediastinum and subcutaneous emphysema does not always indicate Boerhaave's syndrome: report of six cases. Surg Today.

[24] Dejene S, Ahmed F, Jack K, Anthony A (2013). Pneumothorax, music and balloons: a case series. Ann Thorac Med.

[25] Macklin MT, Macklin CC (1944). Malignant interstitial emphysema of the lungs and mediastinum as an important occult complication in many respiratory diseases and other conditions: an interpretation of the clinical literature in the light of laboratory experiment. Medicine.

[26] Grant K, Beckert L (2019). Music as a risk factor for primary spontaneous pneumothorax. NZ Med J.

[27] Noppen M, Verbanck S, Harvey J, Van Herreweghe R, Meysman M, Vincken W, Paiva M (2004). Music: a new cause of primary spontaneous pneumothorax. Thorax.

[28] Johnson DB, Tierney MJ, Sadighi PJ (2004). Kapalabhati pranayama: breath of fire or cause of pneumothorax? A case report. Chest.

[29] Ghisalberti M, Guerrera F, De Vico A (2020). Age and clinical presentation for primary spontaneous pneumothorax. Heart Lung Circ.

[30] Tschopp JM, Bintcliffe O, Astoul P (2015). ERS task force statement: diagnosis and treatment of primary spontaneous pneumothorax. Eur Respir J.

[31] Hendin A, Koenig S, Millington SJ (2020). Better with ultrasound: thoracic ultrasound. Chest.

[32] Almajid FM, Aljehani YM, Alabkary S, Alsaif HS (2019). The accuracy of computed tomography in detecting surgically resectable blebs or bullae in primary spontaneous pneumothorax. Radiol Med.

[33] Noppen M, De Keukeleire T (2008). Pneumothorax. Respiration.

[34] Iqbal B, Hallifax R, Rahman NM (2025). Pneumothorax: an update on clinical spectrum, diagnosis and management. Clin Med (Lond).

